# On the Validity and Benefit of Manual and Automated Drift Correction in Reading Tasks

**DOI:** 10.3390/jemr18030017

**Published:** 2025-05-09

**Authors:** Naser Al Madi

**Affiliations:** Department of Computer Science, Colby College, Waterville, ME 04901, USA; nsalmadi@colby.edu

**Keywords:** eye tracking, reading, correction, drift, automated-algorithms, distortion

## Abstract

Drift represents a common distortion that affects the position of fixations in eye tracking data. While manual correction is considered very accurate, it is considered subjective and time-consuming. On the other hand, automated correction is fast, objective, and considered less accurate. An objective comparison of the accuracy of manual and automated correction has not been conducted before, and the extent of subjectivity in manual correction is not entirely quantified. In this paper, we compare the accuracy of manual and automated correction of eye tracking data in reading tasks through a novel approach that relies on synthetic data with known ground truth. Moreover, we quantify the subjectivity in manual human correction with real eye tracking data. Our results show that expert human correction is significantly more accurate than automated algorithms, yet novice human correctors are on par with the best automated algorithms. In addition, we found that human correctors show excellent agreement in their correction, challenging the notion that manual correction is “highly subjective”. Our findings provide unique insights, quantifying the benefits of manual and automated correction.

## 1. Introduction

Eye tracking has been an important methodology in the study of reading natural language text [1], source code [2], and music [3]. In reading research, eye movement is often studied on the word level, requiring higher temporal and spatial accuracy. Temporal accuracy is associated with the sampling frequency of the eye tracker, and it indicates the amount of eye movement data recorded relative to the data lost during recording [4]. Spatial accuracy, on the other hand, is measured in degrees of visual angle between the real fixation position and the detected fixation position. The variation between the detected gaze location and the actual gaze location is called ***drift*** [5]. Many factors could influence data quality in eye tracking studies, including the following:

1. **Eye-tracking equipment:** The type resolution and frequency of the eye tracker can influence drift [4,6]. For example, remote eye trackers are more susceptible to error compared to tower-mounted eye trackers. In addition, the resolution of the eye tracker camera could limit the detection of pupil movement. Eye tracker frequency determines the smallest eye movement event that can be detected, and if the frequency is low, some samples could be lost, resulting in inaccuracy in the detected fixation position.
2. **Pupil-size changes:** Most camera-based eye-trackers rely on pupil corneal reflection in detecting the fixation position on the screen. Recently, Hooge et al. [7] found that changes in the pupil size significantly affect the accuracy of the detected fixation position.
3. **Participant differences:** Holmqvist et al. [8] reported that differences and variations in physiology, neurology, and psychology between participants may influence data quality depending on how the eye model used by the system accounts for these variations. Typically, eye tracking systems rely on models that interpret eye position and movement based on IR-light reflection. These models often assume certain standard eye characteristics, but differences in the physical structure of the eye and surrounding anatomy such as eye color, wearing glasses, contact lenses, eyelid shape, eyelash interference can deviate from model assumptions. Similarly, children and adults may show different patterns of oculomotor control, reflecting neurological development [9]. In addition, differences in attention, motivation, or mental state can translate to variations in eye tracking data quality. For example, a fatigued participant may blink more, have decreased saccadic velocity, or have longer reaction times [10,11].
4. **Operator skill:** More experienced eye tracker operators are often able to record data with higher accuracy [8].
5. **Eye and eye-tracker positioning:** Adjusting the distance from the eye tracker, infra-red intensity, and the positioning of the monitor and eye tracker can play a role in the quality of the data [8]. Generally, eye trackers measure the angle of gaze to estimate the gaze position on the screen, and this angle is dependent on the distance between the participant and screen and the dimensions of the screen. Most eye trackers have a limit to the range of eye movement angles they can accurately track. For example, many commercial eye trackers can reliably track movements within ±20–30 degrees of the center (gaze straight ahead).
6. **Calibration:** Difficulties in calibration [12], in addition to calibration degradation over time, result in lower data quality.
7. **Head movement:** Free head movement during the experiment can negatively influence calibration accuracy, and subsequently, data quality [12].
8. **Lighting changes:** Changes in room lighting could influence pupil size and the detection of corneal reflection resulting in lowered accuracy [4,12].

**Figure 1:**
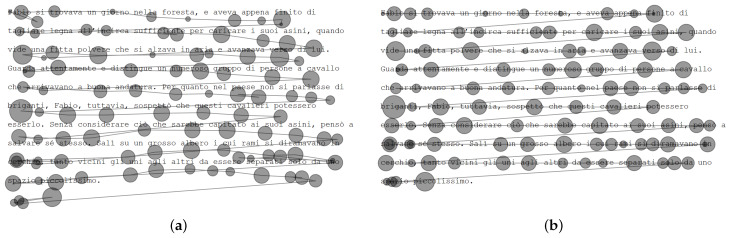
Example trial showing fixations drift (data from Carr et al. [13]). (**a**) Trial before correction (showing drift). (**b**) Trial after correction.

During reading, drift can move fixations from one word to another or one line to another, influencing research findings. Figure 1 shows a real eye tracking trial from Carr et al. [13], where the position of fixations drifts from the intended words, illustrating how drift can be detrimental to recording quality. Poor data quality can influence key experimental measures, leading to the misidentification of group differences and eye-movement patterns [14,15]. In reading research in particular, it was found that poor quality in fixation positions can falsely produce nonexistent eye-movement effects [16].

Drift can be identified and manually corrected, which is often considered the most accurate approach to correcting eye tracking data in reading [5,13,17,18]. Yet manual correction is often considered “subjective” and “not necessarily reproducible” [19,20]. Therefore, one or two human correctors often correct the data collaboratively, relying on a visualization similar to Figure 1 to move fixations to the intended word. This process relies on the experience of the corrector, and two correctors share this responsibility to account for subjective differences between correctors (subjectivity).

Manual correction is time-consuming and is considered a laborious task, even for a relatively small number of eye tracking trials. Therefore, many automated algorithms have been proposed over the years [12,13,18,21,22,23,24,25,26,27,28]. Each automated algorithm is a heuristic based on specific assumptions about eye tracking data and distortion patterns, and algorithms are assessed against a gold-standard dataset corrected manually by human experts. By comparing the correction of the algorithm to the manual correction of human experts, the accuracy of the algorithm is calculated. While automated algorithms continue to improve in accuracy, manually corrected data is still considered the gold standard. An objective comparison of the accuracy of manual and automated correction has not been conducted before, and the degree of subjectivity in manual correction is not entirely quantified and often accepted anecdotally. Understandably, measuring the accuracy of manual correction objectively is challenging considering that research often relies on manual correction as ground truth.

In this paper, we compare the accuracy of the manual and automated correction of eye tracking data in reading tasks through a novel approach that relies on synthetic data with known ground truth. Moreover, we measure subjectivity and agreement in manual human correction with real eye tracking data. Our research questions are as follows:

- RQ1: How much of an accuracy benefit is gained by manual correction over automated correction?
- RQ2: How often do manual correctors disagree on correcting eye tracking data in reading tasks?

Our novel approach to assess the accuracy of manual correction consists of generating a set of synthetic eye tracking trials, subsequently introducing distortion to them before they are given to novice and experienced human correctors. By comparing the human-corrected trials to the original trials before distortion was introduced, we can objectively measure the accuracy of human correction. Similarly, we can correct the same distorted trials with the automated correction algorithm and measure the accuracy of automated correction algorithms.

Subjectivity in manual correction can be measured by examining the agreement between different correctors or how often different correctors apply the same correction to a given fixation. Also, we measure the Intra-Class Correlation Coefficient (ICC3) [29] to assess the consistency or agreement of measurements made by multiple raters.

Relying on data from four real eye tracking datasets to answer these research questions, we highlight the trade-offs between manual and automated correction methods, quantifying the benefits and disadvantages of each correction approach.

## 2. Materials and Methods

This section begins with an overview of the manual correction data from Al Madi et al. [30] on which we rely on in this study. Then, we present our methodology for collecting data from automated correction algorithms, and finally, we present the details of our analyses.

The manual correction data were drawn from Al Madi et al. [30]. The previous study compared manual correction to an assisted (semi-automated) approach to correcting eye tracking data through a tool with a graphical user-interface. While the previous study focused on presenting and validating the assisted-correction approach, in this study, we focus on comparing manual correction to automated correction to understand and quantify the trade-offs between the two correction approaches. This study was approved by the Institutional Review Board (IRB) of Colby College (#2023-064).

### 2.1. Manual Correction

This study utilizes manual correction data from Al Madi et al. [30], and we review the relevant details about the data in this section. Refer to the original study for a more comprehensive presentation of the experiment and its details.

**Figure 2:**
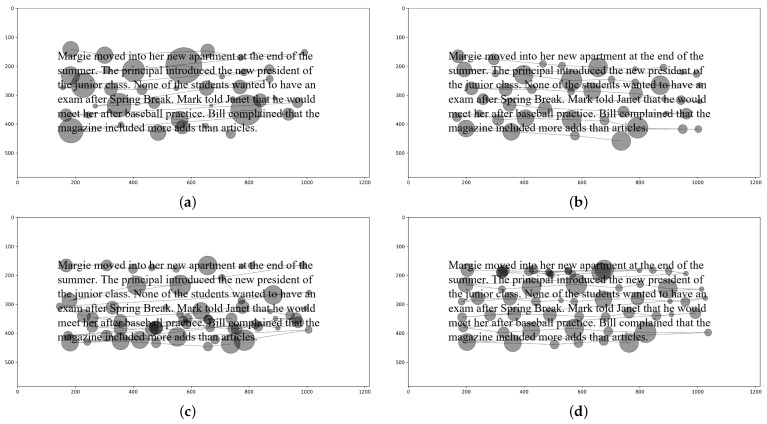
Synthetic eye tracking data showing (**a**) noise, (**b**) slope, (**c**) shift, and (**d**) offset distortions in reading (from Al Madi et al. [30]).

The data from Al Madi et al. [30] comes from 14 participants who were adult college students. Five are female and nine are male, and they participated voluntarily and were awarded a $20 gift card after completing the experiment. Participants were given real and synthetic eye tracking trials to correct. Eighteen synthetic trials included varying degrees of noise, slope, shift, and offset distortions, along with trials containing within-line and between-line regressions. For consistency, we use the same names and implementation presented by [13] in this paper. Figure 2 shows four sample trials.

According to [13,18], noise describes a situation where a subset of fixations experience a distortion on the y-axis, resulting in some fixations drifting above the text and some below the text. Slope describes a situation where fixations experience drift on one side of the screen due to calibration error, leaving fixations on one side intact while fixations on the other side drift below (or above) the text. Shift describes the same phenomenon on the vertical axis, where the first line might be intact and other lines progressively experience drift with the maximum drift at the last line. Offset describes a consistent drift affecting all fixations in the same proportion. Within-line regressions describes a reading behavior, where the eyes make a jump to a previous word on the same line, and similarly, between-line regressions describe jumps to a previous line.

Initially, the synthetic trials were generated without distortions, then distortions and regressions were introduced. This allows us to compare the human-corrected trial to the original trial (before distortions) to assess correction accuracy. Using these synthetic data allows for a unique and accurate way of objectively assessing the accuracy of manual correction.

As described by Al Madi et al. [30], the synthetic data are generated with a level of realism in terms of fixation durations and typical reading phenomena such as word skipping. Fixation duration is proportional to word length in terms of character spaces, and short words are more likely to be skipped. Each distortion and regression type is captured at varying magnitudes, resulting in trials with low, medium, and high levels of distortion.

The ErrorNoise generator in Algorithm 1 introduces vertical noise to a list of eye-tracking fixation points to simulate measurement inaccuracies or distortions. Each fixation is represented as a triplet containing the *x*-coordinate, *y*-coordinate, and fixation duration. The algorithm iterates over each fixation and perturbs the *y*-coordinate by adding a random value (positive or negative) drawn from a Gaussian distribution with a mean of 0 and standard deviation equal to a user-specified parameter $y_{noise}$. The *x*-coordinate and duration remain unchanged. The modified fixation is then stored in a result list, which is returned at the end.
**Algorithm 1** ErrorNoise: Add vertical Gaussian noise to fixations**Require:** $y_{noise}\in R$: standard deviation of vertical noise **Require:** $fixations\in R^{n\times 3}$: list of fixation triplets [x,y,duration] **Ensure:** $results\in R^{n\times 3}$: list of distorted fixations
1:results← empty list2:**for** i=0 **to** n−1 **do**3:   x←fixations[i][0]4:   y←fixations[i][1]5:   duration←fixations[i][2]6:   $distorted\_y\leftarrow y+N(0,y_{noise})$7:   Append [x,distorted_y,duration] to results8:**end for**9:**return** results

The ErrorSlope generator in Algorithm 2 simulates a distortion in eye-tracking data by altering the vertical (*y*) coordinate of each fixation according to its horizontal (*x*) displacement from the first fixation. It takes as input a list of fixation triplets [x,y,duration] and a slope factor that controls the degree of vertical drift across the horizontal axis. The algorithm begins by storing the *x*-position of the first fixation, which serves as a reference. For each fixation, it calculates the horizontal offset from this reference and uses it to compute a vertical adjustment proportional to the given slope factor. This modified *y*-coordinate is combined with the original *x*-coordinate and duration, and the adjusted fixation is stored. The result is a list of fixations with a consistent vertical trend applied across their *x*-positions.

The ErrorShift generator in Algorithm 3 introduces a vertical distortion to the eye-tracking fixation coordinates by shifting each fixation’s *y*-value based on its vertical distance from a reference line (typically the top line). It accepts as input a list of fixation triplets [x,y,duration], a list of line y-coordinates, and a shift factor that determines the magnitude of distortion. The algorithm begins by computing the line height as the vertical distance between the first two lines. Then, for each fixation, it calculates how far the fixation’s *y*-value is from the first line and applies a scaled distortion proportional to that distance and the shift factor. This adjusted *y*-value is combined with the original *x*-value and duration, and the distorted fixation is stored. The resulting output is a list of fixations with a smooth vertical shift that increases with distance from the top of the screen.
**Algorithm 2** ErrorSlope: Apply linear vertical distortion based on the x-position**Require:** slope_factor∈R: vertical slope distortion factor **Require:** $fixations\in R^{n\times 3}$: list of fixation triplets [x,y,duration] **Ensure:** $results\in R^{n\times 3}$: list of slope-distorted fixations
  1:results← empty list  2:first_x←fixations[0][0]  3:**for** i=0 **to** n−1 **do**  4:   x←fixations[i][0]  5:   y←fixations[i][1]  6:   duration←fixations[i][2]  7:   $adjusted\_y\leftarrow y+\left(\frac{x-first\_x}{100}\cdot slope\_factor\right)$  8:   Append [x,adjusted_y,duration] to results  9:**end for**10:**return** results

**Algorithm 3** ErrorShift: Apply vertical shift distortion relative to the line positions**Require:** y_shift_factor∈R: vertical shift distortion factor **Require:** $line\_ys\in R^{m}$: list of line y-coordinates **Require:** $fixations\in R^{n\times 3}$: list of fixation triplets [x,y,duration] **Ensure:** $results\in R^{n\times 3}$: list of vertically shifted fixations
  1:results← empty list  2:line_height←line_ys[1]−line_ys[0]  3:**for** i=0 **to** n−1 **do**  4:   x←fixations[i][0]  5:   y←fixations[i][1]  6:   duration←fixations[i][2]  7:   distance←|y−line_ys[0]|  8:   $adjusted\_y\leftarrow y+\left(\frac{distance}{line\_height}\cdot y\_shift\_factor\right)$  9:   Append [x,adjusted_y,duration] to results10:**end for**11:**return** results

The ErrorOffset generator in Algorithm 4 introduces a uniform distortion to the eye-tracking fixation coordinates by applying fixed horizontal and vertical offsets to each fixation. It accepts a list of fixations represented as triplets [x,y,duration], along with scalar values x_offset and y_offset, indicating how much to shift each fixation in the x- and y-directions, respectively. The algorithm iterates through each fixation, adds the offsets to the x- and y-values, and preserves the original duration. The result is a list of systematically displaced fixations that simulate a consistent calibration error or systematic drift in recorded eye-tracking data.

The BetweenLineRegression generator in Algorithm 5 generates synthetic fixations based on the layout of words (AOIs) in a reading stimulus. For each word, it computes a fixation slightly to the left of the word’s center (at roughly one-third width) and vertically centered on the line, with added jitter to simulate natural variability around the optimal viewing position. Fixation durations are scaled proportionally to the word width. Additionally, the algorithm introduces between-line regressive fixations that jump backward to earlier words on previous lines based on a user-defined probability. Candidate indexes for regressions are randomly selected, and the algorithm ensures that at least one regression occurs if the regression probability is non-zero. When a regression is triggered, the algorithm attempts to select a valid target on a different line by retrying up to ten times. This simulates more realistic regressions in reading patterns while maintaining the spatial and temporal plausibility of the fixation data.
**Algorithm 4** ErrorOffset: Apply fixed x/y offset to the fixation coordinates**Require:** x_offset∈R: horizontal offset **Require:** y_offset∈R: vertical offset **Require:** $fixations\in R^{n\times 3}$: list of fixation triplets [x,y,duration] **Ensure:** $results\in R^{n\times 3}$: list of offset fixations
  1:results← empty list  2:**for** i=0 **to** n−1 **do**  3:   x←fixations[i][0]  4:   y←fixations[i][1]  5:   duration←fixations[i][2]  6:   adjusted_x←x+x_offset  7:   adjusted_y←y+y_offset  8:   Append [adjusted_x,adjusted_y,duration] to results  9:**end for**10:**return** results

**Algorithm 5** BetweenLineRegression: Generate fixations with between-line regressions**Require:** aois_list: list of Areas-Of-Interest (AOIs) **Require:** regression_probability∈[0,1] **Ensure:** fixations: list of synthetic fixations
  1:Select regression_indexes probabilistically from indices >2  2:**if** no regression_indexes and regression_probability>0 **then**  3:    Add one random regression index from valid range  4:**end if**  5:index←0  6:**while** index<len(aoi_list) **do**  7:    Extract x,y,width,height,line from aoi_list[index]  8:    $fixation\_x\leftarrow x+\frac{width}{3}+\mathrm{jitter}$, $fixation\_y\leftarrow y+\frac{height}{2}+\mathrm{jitter}$  9:    $duration\leftarrow 100+\left(\frac{width}{15}\times 40\right)$10:   Append [fixation_x,fixation_y,duration] to fixations11:   **if** index∈regression_indexes **then**12:       Try up to 10 times to select a prior index on a different line13:       If successful, set index←priorindex and **continue**14:   **end if**15:   index←index+116:**end while**17:**return** fixations

The WithinLineRegression generator in Algorithm 6 simulates eye fixations along lines of text, introducing within-line regressions with a specified probability. This generator is mostly identical to the BetweenLineRegression generator; the only difference is that it guarantees a regression within the same line of text instead of a previous line. When a regression index is reached, the algorithm attempts to jump back to a previous word on the same line, simulating a within-line regression. This process continues returning a list of generated fixations that emulate realistic reading behavior with occasional regressive eye movements confined to the same line.

**Figure 3:**
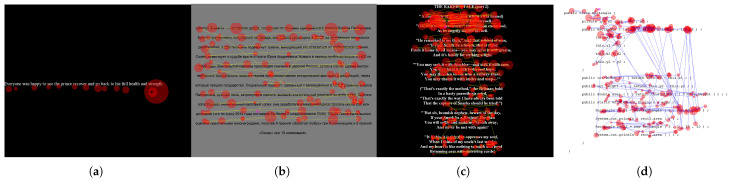
Sample trials from the four datasets. (**a**) AlMadi2018; (**b**) MET dataset; (**c**) GazeBase dataset; (**d**) EMIP dataset.

In addition to the synthetic data, 18 real-data trials come from four datasets, five trials from Al Madi and Khan [31], five from the Multilingual Eye Tracking (MET) dataset [32], four from GazeBase [33], and four from the Eye Movement In Programming (EMIP) dataset [34]. Figure 3 shows sample trials from each dataset, which represent diverse reading experiments reflecting differences in language, number of lines of text, font height, spacing, and eye tracking frequency. The set of real-data trials is used in measuring subjectivity with highly realistic and representative data.
**Algorithm 6** WithinLineRegression: Generate fixations with within-line regressions**Require:** aoi_list: list of Areas-Of-Interest (AOIs) **Require:** regression_probability∈[0,1] **Ensure:** fixations: list of generated fixations
  1:Select regression_indexes probabilistically from indices >2  2:**if** regression_indexes is empty and regression_probability>0 **then**  3:    Add one random regression index to ensure coverage  4:**end if**  5:index←0  6:**while** index<len(aoi_list) **do**  7:    Extract x,y,width,height,line from aoi_list[index]  8:    Compute fixation location with small horizontal/vertical jitter  9:    $duration\leftarrow 100+\left(\frac{width}{15}\times 40\right)$10:   Append [fixation_x,fixation_y,duration] to fixations11:   **if** index∈regression_indexes **then**12:       Try up to 10 times to pick prior index on the same line13:       **if** successful **then**14:           index← chosen prior index15:           **continue**16:       **end if**17:     **end if**18:     index←index+119:**end while**20:**return** fixations

In the original study by Al Madi et al. [30], participants corrected half of the trials manually and half using the assisted approach presented in the article. For the purpose of this study, we focus only on trials that were corrected manually, excluding trials that were corrected using the assisted technique. In addition, the original study relied on self-reporting for experience, while here, we rely on the overall performance of each participant. There was a clear gap in the performance of participants, resulting in labeling ten participants as novices and four as experts. More specifically, we took the mean accuracy of each participant in addition to their self-reported experience to label participants as either experts or novices. When the overall accuracy of a participant exceeded 90%, they were entered into the advanced group. All but one person in the experienced group reported being experienced in eye tracking data correction. In other words, participant performance matched self-reported experience with 12 out of 14 participants (one novice and one expert).

### 2.2. Automated Correction

For comparison with manual correction, the same synthetic trials containing distortion are given to nine automated correction algorithms. The accuracy of each algorithm is assessed by comparing the corrected trial to the original synthetic trial before distortion was introduced.

The automated correction algorithms fall into various categories based on the heuristic strategy that guides their correction. As described by Carr et al. [13], positional algorithms include the *Attach* algorithm, which assigns each fixation to its closest text line, and the *Chain* algorithm, which groups consecutive fixations and assigns them to the line closest to the mean of their y-values [13,35]. Relative positional approaches such as *Cluster* use k-means clustering to group fixations with similar y-values, while *Merge* progressively combines fixation sequences until they match the number of text lines [35,36]. The *Regress* algorithm treats fixations as a cloud of points and fits regression lines [37], whereas *Stretch* minimizes the alignment error between fixation points and text lines through adjustments in offsets and scaling factors [20]. Sequential algorithms like *Segment* divide fixation sequences into discrete subsequences mapped linearly from top to bottom [21], while *Warp* employs Dynamic-Time Warp to minimize Euclidean distances between fixation positions and word centers [13].

The Attach algorithm, as implemented by Carr et al. [13], takes as input a list of 2D fixation coordinates (fixation_XY) and a list of y-values corresponding to horizontal text lines (line_Y). Its purpose is to align each fixation vertically with the nearest line, effectively “snapping” the eye movement data to the most probable reading line. For each fixation point, it computes the vertical (y-axis) distance to all lines in line_Y, identifies the line with the minimum absolute difference, and it replaces the fixation’s y-coordinate with that of the closest line. This process ensures that all fixations are vertically aligned to the most likely text line.

Here, we present a more optimized version of the Attach algorithm (Algorithm 7), which improves efficiency by using binary search to find the nearest text line for each fixation. Assuming the array of line positions (*line_Y*) is sorted, the algorithm iterates through each fixation in *fixation_XY*, retrieves its y-coordinate, and uses binary search to find the index of the closest value in *line_Y*. It then replaces the fixation’s y-value with that of the nearest line. Because binary search reduces the search from linear time 𝒪(m) to logarithmic time 𝒪(logm), the overall time complexity improves from 𝒪(n·m) to 𝒪(n·logm), where *n* is the number of fixations and *m* is the number of lines of text. This optimization improves runtime, especially for large datasets.
**Algorithm 7** Optimized Attach fixations to the closest line (optimized with binary search)**Require:** $fixation\_XY\in R^{n\times 2}$, array of fixation coordinates **Require:** $line\_Y\in R^{m}$, sorted array of y-coordinates of lines **Ensure:** Modified fixation_XY with updated y-values
1:n← length of fixation_XY2:**for** fixation_i=0 **to** n−1 **do**3:   y←fixation_XY[fixation_i,1]4:   line_i← Index of value in line_Y closest to *y* (using binary search)5:   fixation_XY[fixation_i,1]←line_Y[line_i]6:**end for**7:**return** fixation_XY


The *Chain* algorithm [35], as implemented by Carr et al. [13], groups sequences of temporally and spatially close eye fixations into chains and aligns them to the nearest text line. It begins by computing the horizontal and vertical distances between consecutive fixations. A new chain is initiated whenever the distance between fixations exceeds either a horizontal threshold (x_thresh) or a vertical threshold (y_thresh), which indicates a transition between reading units such as words or lines. For each chain of fixations, the algorithm calculates the average y-coordinate and finds the closest line in *line_Y* to that average. It then aligns all y-values within the chain to the y-coordinate of that line, effectively snapping entire chains rather than individual fixations to their most likely text line.

The optimized version of the *Chain* algorithm, presented here in Algorithm 8, improves performance by using binary search to find the closest line for each chain of fixations. First, the algorithm calculates the pairwise distances between consecutive fixations in both x- and y-dimensions. It identifies the boundaries of fixation chains based on whether these distances exceed predefined thresholds (x_thresh and y_thresh), which typically signal line or word transitions. For each resulting chain, it computes the mean y-coordinate and uses binary search on the sorted array of line positions (line_Y) to efficiently find the nearest line. All fixations in the chain are then aligned to that line’s y-coordinate. By replacing a linear search with binary search, the algorithm reduces the complexity of line assignment from 𝒪(k·m) to 𝒪(k·logm), where *k* is the number of chains. *n* is the number of fixations and *m* is the number of lines of text.
**Algorithm 8** Optimized Chain fixations to the nearest line**Require:** $fixation\_XY\in R^{n\times 2}$, array of fixation coordinates **Require:** $line\_Y\in R^{m}$, sorted array of line y-positions **Require:** x_thresh,y_thresh – spatial thresholds for chaining **Ensure:** Modified fixation_XY with grouped y-values aligned to lines
  1:n← length of fixation_XY  2:dist_X←|distancesbetweenconsecutivefixationcoordinatesinthex|  3:dist_Y←|distancesbetweenconsecutivefixationcoordinatesinthey|  4:end_chain_indices← indices where dist_X>x_thresh or dist_Y>y_thresh  5:Increment all end_chain_indices by 1 and append *n*  6:start_of_chain←0  7:**for all** end_of_chain∈end_chain_indices **do**  8:    mean_y←mean(fixation_XY[start_of_chain:end_of_chain,1])  9:    line_i←BinarySearchClosest(line_Y,mean_y)10:    fixation_XY[start_of_chain:end_of_chain,1]←line_Y[line_i]11:    start_of_chain←end_of_chain12:**end for**13:**return** fixation_XY


The *Cluster* algorithm, which is based on Ref. [35] and implemented by the authors in [13], groups fixations into *m* vertical clusters, where *m* is the number of expected text lines (i.e., the length of line_Y). The algorithm applies KMeans clustering to the y-values of the fixations, resulting in *m* clusters corresponding to groups of fixations that likely fall on the same line of text. Each cluster’s mean y-coordinate is then computed, and the clusters are sorted by vertical position from top to bottom. After sorting, the algorithm matches the topmost cluster to the topmost line in line_Y, the second-highest cluster to the second line, and so on. Each fixation is then reassigned the y-coordinate of the matched line. This approach depends on the quality of clustering and the assumption that the number of lines is known in advance. The algorithm runs in approximately 𝒪(n·m·t) time, where *t* is the number of KMeans iterations.

The *Merge* algorithm, based on [36] and implemented by [13], is a multi-phase heuristic designed to align eye fixation sequences to specific text lines. The algorithm begins by identifying natural breaks between fixations, using horizontal (X) backtracking regressions or large vertical (Y) jumps to split the sequence into initial candidate groups. These groups, called “sequences”, are then iteratively merged across four phases with increasingly relaxed constraints. In each phase, pairs of sequences are evaluated for potential merging based on their linear fit—the slope (gradient) and residual error of a regression line through their combined fixation coordinates. A merge is accepted if the pair satisfies the gradient and error thresholds, except in the final phase where these constraints are ignored. The goal of merging is to reduce the number of fixation sequences to match the number of text lines. Once enough merges have occurred, the algorithm computes the mean y-position of each sequence, sorts them, and aligns each group to the closest corresponding line. In practice, the algorithm runs in approximately $𝒪(n^{2})$ time (average time complexity).

The *Regress* algorithm is a probabilistic approach to aligning eye fixations to lines of text by estimating the most likely mapping using a fitted regression model [37]. The core idea is that fixations may be misaligned vertically due to systematic distortions, such as screen-viewing angles or calibration errors. To correct this, the algorithm searches for the optimal parameters of a simple linear transformation—a slope, an offset, and a noise spread—which are constrained to fall within user-specified bounds. These parameters are searched using a gradient-based optimization routine (*minimize*) that aims to maximize the likelihood (or minimize the negative log-likelihood) that the observed vertical fixation positions were drawn from Gaussian distributions centered on the predicted positions for each line. Each fixation’s predicted y-coordinate is calculated from its x-position and the current slope estimate, plus an offset relative to each candidate line. For every line, the algorithm evaluates the log-probability of each fixation coming from that line and assigns each fixation to the line with the highest probability. Once the best-fit parameters are found, the y-positions of fixations are snapped to their most likely lines, producing an alignment that accounts for both linear distortion and reading noise. The algorithm runs in approximately 𝒪(t·n·m) time, where *t* is the number of iterations or function evaluations performed by the optimizer.

The *Segment* algorithm is a heuristic method designed to assign eye fixation coordinates to specific lines of text based on patterns of horizontal eye movement, which are characteristic during reading [21]. It assumes that the most significant horizontal saccades typically correspond to transitions between lines—for example, when a reader reaches the end of one line and moves to the start of the next. The algorithm begins by measuring the horizontal distances between consecutive fixations and ranks the saccades by length. From this ranking, it identifies the (m−1) smallest saccades as probable indicators of line changes, where *m* is the total number of text lines. The algorithm then iterates through the sequence of fixations, assigning each one to a line by adjusting its vertical coordinate to match the corresponding line position. When a fixation index corresponds to one of the identified line change positions, the algorithm increments the current line index to move to the next line. This effectively segments the sequence of fixations across the text lines, assuming that line transitions are marked by smaller horizontal movements relative to within-line reading behavior. The algorithm runs in approximately 𝒪(n+mlogm) time.

The *split* algorithm is designed to correct vertical drift in eye-tracking data by segmenting sequences of fixations into individual reading lines using horizontal movement patterns [13]. It begins by calculating the horizontal distances (saccades) between consecutive fixations. These saccades are clustered into two groups using KMeans clustering, which assumes that one group represents short intra-line fixations and the other represents long sweep saccades that typically occur at line boundaries. By identifying which cluster corresponds to these longer sweep saccades (determined by the cluster with the lower mean), the algorithm determines the indices at which a line transition occurs. Using these indices, the algorithm splits the fixation sequence into line segments. For each segment, it computes the mean vertical position of fixations and aligns all fixations within that segment to the closest line in the provided set of reference line positions (*line_Y*). This approach enables the adaptive correction of drift by leveraging natural horizontal eye movement patterns during reading. The most computationally intensive operation is KMeans clustering on n−1 saccades, leading to an overall time complexity of 𝒪(n) for preprocessing and segmentation, assuming KMeans converges in constant time due to the fixed number of clusters.

The *stretch* algorithm corrects vertical drift in eye-tracking data by globally adjusting the vertical positions of fixations through a linear transformation involving both scaling and offset. It assumes that vertical drift can be approximated by a uniform stretch and shift of fixation y-coordinates [13]. To achieve this, the algorithm defines an objective function that computes transformed y-values using a given scale and offset, and ot aligns each transformed fixation to the nearest line in the reference array *line_Y*. The cost is calculated as the sum of absolute differences between the transformed y-values and their corresponding aligned lines. Using bounded optimization, the algorithm finds the parameters that minimize this cost, constrained within user-defined bounds. Once the optimal scale and offset are determined, the final corrected y-positions are generated by aligning the transformed coordinates to the closest lines. Since each evaluation of the objective function involves all *n* fixations and the number of parameters is constant, the time complexity of the algorithm is 𝒪(n·t), where *t* is the number of optimizer iterations.

The *warp* algorithm corrects vertical drift in eye-tracking data by aligning each fixation to the most likely line of text based on its proximity to corresponding words [13]. It employs Dynamic Time Warping (DTW) to establish an optimal alignment between the sequence of fixations and the sequence of word positions (*word_XY*). DTW constructs a cost matrix using pairwise Euclidean distances between fixations and words and finds a minimum-cost path that allows for non-linear alignment to accommodate variations in reading pace. Once this path is obtained, each fixation is associated with one or more words, and its y-coordinate is updated to the mode of the y-values of those associated words, effectively snapping it to the most probable reading line. This approach ensures a robust correction that is tolerant to noise while maintaining alignment with the structure of the text. The overall time complexity of the algorithm is 𝒪(n·m), where *n* is the number of fixations and *m* is the number of words.

### 2.3. Analyses

Our first research question compares the accuracy of manual correction with automated algorithms on the synthetic data. Here, we focus on the synthetic data exclusively, to avoid relying on human correction as the ground truth leading to circular bias. We compare manual and automated corrections to the original trials before distortion was introduced. This way, the comparison is objective and the ground truth data are completely reliable.

Accuracy in the context of correcting eye tracking data in reading tasks is measured as the percentage of fixations that are returned to the same line they were on before distortion was introduced. In other words, if fixation_n was on line 1, then after distortion was introduced, it was moved to another line or an empty space between the line, and a valid correction should return that fixation to line 1. The correction accuracy for a given trial is calculated by counting the number of fixations that were returned to their original lines over the total number of fixations in the trial.

The second research question is concerned with subjectivity in manual correction. Subjectivity between human correctors would be reflected in applying different corrections to the same fixation, and therefore, it is the opposite of agreement. In other words, high subjectivity would result in low agreement between correctors and vice versa. Therefore, we measure agreement between correctors as the percentage of fixations where correctors assigned a given fixation to the same line.

Considering that human correctors corrected the same set of trials, we can give each fixation in each trial a unique ID. Then, we can examine the corrected trials from each participant, and for each fixation, we can record the line number it was assigned to in that trial. When all corrected trials from all correctors are processed, we can go over fixations one at a time and measure how often correctors assigned the fixation to the same line. For example, if three out of four correctors assigned a given fixation to the same line, then the agreement for that fixation is 75%. The overall agreement between correctors is the mean of the agreements on all fixations.

For the agreement, we focus on the corrected trials from human correctors without being concerned with accuracy. The goal is to measure how often correctors agree, not if correctors made the right correction or not. Therefore, we measure agreement over the real data for generalization.

Furthermore, we approach the agreement question statistically. Studies often use Cohen’s Kappa [38] to assess the agreement between two raters, while in our study, we had 14 raters and therefore we rely on intra-class correlation to assess agreement. The Intra-Class Correlation Coefficient (ICC3) is a statistical measure used to assess the consistency or agreement of measurements made by multiple raters or instruments on the same subjects when a fixed set of raters is used for all observations [29]. ICC3 is particularly sensitive to both systematic and random errors, as it treats differences between raters as part of the measurement error rather than a random factor. Its value ranges from −1 to 1, where higher values indicate stronger agreement. Typically, ICC3 is interpreted as follows: values above 0.75 indicate excellent reliability, values between 0.50 and 0.75 suggest moderate reliability, and values below 0.50 reflect poor reliability.

## 3. Results

In this section, we start by comparing the accuracy of manual correction to automated correction over synthetic data. Then, we assess subjectivity by examining the agreement among correctors in correcting real eye tracking data. In both accuracy and subjectivity, we explore the differences between experienced and novice correctors.

### 3.1. Accuracy

**Figure 4:**
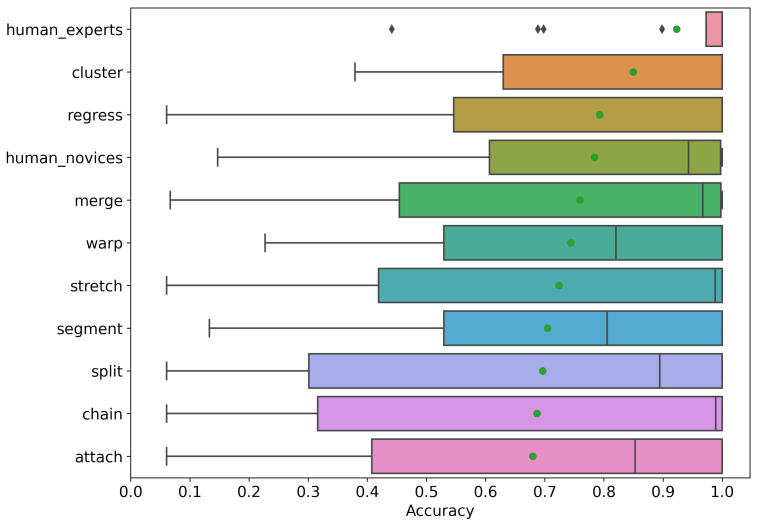
Manual and automated correction accuracy. Green dot is the mean.

Here, we compare the accuracy of manual correction to automated algorithms in correcting synthetic trials with known ground truth. Figure 4 shows the mean accuracy (green dot), median accuracy (vertical line), and standard deviation (error bars) for human novices, experts, and algorithms. As seen in the figure, the accuracy of automated algorithms ranged from 67% (Attach) to 84% (Cluster), while the mean manual correction of novices was 78% and of experts was 92%. Moreover, the best-performing expert had an accuracy of 95%, which is 11% better than the best automated algorithm.

A Mann-Whitney U test was conducted to compare the correction accuracy between novices and experts. The results indicated a statistically significant difference, U = 815.0, *p* = 0.0018. The negative effect size, Cohen’s d = −0.63, suggests a medium effect, indicating that experts performed substantially better than novices in correction accuracy. These findings highlight a notable distinction in performance between the two groups.

### 3.2. Subjectivity

Here, we assess the subjectivity of novices and experts in correcting real data. We start by measuring the agreement between correctors as the percentage of fixations in which correctors assign a given fixation to the same line. We found that, on average, novices agree on the position of 88.5% of fixations, while experts agree on 98.8% fixations. This suggests that experts show very high agreement, indicating low subjectivity in their correction.

Moreover, we approach the question of subjectivity from the inter-rater agreement direction. Studies often use Cohen’s Kappa [38] to assess the agreement between two raters, while in our study we had 14 raters; therefore, we rely on the Intra-Class Correlation Coefficient (ICC3) to assess agreement. The ICC3 is particularly sensitive to both systematic and random errors, as it treats differences between raters as part of the measurement error rather than a random factor [29]. Its value ranges from −1 to 1, where higher values indicate stronger agreement. Typically, the ICC3 is interpreted as follows: values above 0.75 indicate excellent reliability, values between 0.50 and 0.75 suggest moderate reliability, and values below 0.50 reflect poor reliability.

Calculating human corrector agreement in multi-line trials in Table 1, we generally find that correctors agree substantially (mean ICC = 0.71), with a few exceptions that seem to reflect challenging trials. These low agreement real-data trials contained many fixations and high distortion. Moreover, the table shows a very small probability that agreement was due to chance, as reflected by the *p*-value column. These results suggest that human correctors have a high agreement in correcting eye tracking data, indicating low subjectivity.

### 3.3. Summary

In our investigation of manual and automated correction accuracy, we found that automated algorithms ranged in accuracy from 67% to 84%, while novice manual correctors on average achieved 78% accuracy and expert correctors 92%. The best-performing expert had an average accuracy of 95%, surpassing the best automated algorithm by 11%. Statistical analysis confirmed that experts performed significantly better than novices in correction accuracy (U = 815.0, *p* = 0.0018), with a medium effect size (Cohen’s d = −0.63).

Regarding subjectivity, experts demonstrated a very high agreement rate in correcting fixations (98.8%), and agreement among novices was high too (88.5%). An analysis of inter-rater agreement (ICC) showed strong consistency among human correctors, with an average ICC of 0.71, suggesting near-excellent agreement in eye-tracking correction.

## 4. Discussion

Our research investigates the validity of certain widely accepted notions about manual and automated correction in reading experiments. To date, there has been no objective comparison of the accuracy of manual corrections versus automated algorithms. Additionally, prior studies such as [19,20,39] describe manual correction as “subjective” and challenging to reproduce, but the level of subjectivity has never been studied objectively. In addition, researchers often report the frequency and maximum accuracy of the eye tracking equipment as an indicator of the quality of the data, but the correlation between eye-tracker frequency and drift is not fully understood.

Therefore, comparing the accuracy of manual and automated correction can help researchers understand and balance the trade-offs between correction time and accuracy. Moreover, knowing how subjective human correctors are, we can quantify a potential threat to the validity of manual correction, if there is one.

We start with asnwering our first research question, **RQ1: How much of an accuracy benefit is gained by manual correction over automated correction?** Our results show that manual correction by experts is substantially better than the best automated algorithm. The best human corrector had an average accuracy of 95%, exceeding the best automated algorithm by 11%. Therefore, manual correction, for the time being, remains the most accurate method of correcting eye tracking data in reading tasks. Until automated algorithms take the lead, expert human correction is the gold standard.

Additionally, we found that the best automated algorithms approximate and sometimes surpass the manual correction of novices. While this result seems to suggest that the accuracy of automated algorithms is somewhat better than expected, it is important to notice the huge variation in the accuracy of automated algorithms. While the variance in human assessment seems to be influenced by experience (i.e., novices and experts), the variance in automated algorithms appears to be influenced by the alignment between the assumptions made by the algorithm and the conditions of the given trial. For example, some algorithms make assumptions about reading linearity (reading from left to right and top to bottom without jumps back) and their performance is governed by how linear reading is in a given trial. In reading source code, for example, reading patterns are known to be non-linear with significant regressions and progressions. Therefore, a linear algorithm, such as Warp, might not be the most suitable. The results suggest that the choice of the correction algorithm should match the characteristics of the trial being corrected.

Finally, the results seem to adjust our preconceived conceptions on manual and automated correction. In summary, although expert manual correction remains the most accurate, automated algorithms can be a close second as a reliable option for many research experiments. There still remains some overall difficulty in drift correction, that is, in distinguishing systematic drift from the real properties of eye-movement behavior.

We now address our second research question, **RQ2: How often do manual correctors disagree on correcting eye tracking data in reading tasks?** Our results appear to challenge the often-held conception that human correction is substantially subjective and “not necessarily reproducible” [19], especially in the case of expert correctors. Our results show that experts disagree in only 1.2% of the corrected fixations, and even when taking all human correctors as a single group, ICC scores show high agreement. Claims of high subjectivity appear to be inconsistent with our findings. Therefore, our results call for the further exploration of subjectivity in correcting eye tracking data, possibly with more data.

It is worth noting that agreement between human correctors does not necessarily indicate that the applied correction is valid, as the correction may still be biased overall. However, the agreement suggests that human correctors follow similar strategies that lead to applying the same correction.

## 5. Conclusions

In this paper, we compared the accuracy of manual and automated correction of eye tracking data in reading tasks. We present a novel approach to make this comparison possible by relying on synthetic data with known ground truth. Our results represent the first assessment of manual correction with objective ground truth data, granting unique insights and quantifying the benefits of manual and automated correction. Moreover, we present an exploration of subjectivity in manual correction, where previous research claimed that manual correction is highly subjective. Yet, our results show that human correctors, especially experts, had high agreement in their corrections, challenging some of the preconceived opinions on manual correction.

## Figures and Tables

**Table 1:** ICC results for real-data trials. Each row represents a real-data trial from a dataset that was corrected by several participants, and the ICC score represents agreement among correctors.

Trial	Dataset	ICC	F	df1	df2	*p*-Value
024_hindi_single_6_LargeFont	MET	0.66	16.674352	135	945	<0.001
027_russian_multi_6_LargeFont	MET	0.40	6.353907	91	637	<0.001
040_urdu_paragraph2_1_LargeFont	MET	0.36	5.675295	469	3283	<0.001
trial_1_participant_100_TEX_R1S1_bg	GazeBase	0.95	176.530545	342	2394	<0.001
trial_1_participant_102_TEX_R1S1_bg	GazeBase	0.95	176.038158	429	3003	<0.001
trial_2_participant_101_rectangle_java2	EMIP	0.73	23.288324	156	1092	<0.001
trial_2_participant_21_rectangle_java2	EMIP	0.67	15.669201	315	1890	<0.001
034_japanese_multi_5_LargeFont	MET	0.91	42.252772	109	327	<0.001
016_spanish_multi_5_LargeFont	MET	0.24	1.963989	87	174	<0.001
trial_1_participant_101_TEX_R1S1_bg	GazeBase	0.87	34.776527	343	1372	<0.001
trial_1_participant_103_TEX_R1S1_bg	GazeBase	0.98	421.786702	343	1372	<0.001
trial_2_participant_178_rectangle_java2	EMIP	0.68	11.891540	230	920	<0.001
trial_5_participant_60_rectangle_java2	EMIP	0.77	18.535659	254	1016	<0.001
**Mean ICC**	**0.71**	**-**	**-**	**-**	**-**	

## Data Availability

The data and code are available at the following OSF replication package: https://osf.io/2apnm/ (accessed on 23 April 2025).
